# A Historical Review of Gastroschisis: Evolution of Understanding, Diagnosis, and Surgical Management

**DOI:** 10.3390/children13010013

**Published:** 2025-12-20

**Authors:** Mohamad Abi Nassif, Emrah Aydın, Jose L. Peiro

**Affiliations:** 1 The Center for Fetal and Placental Research, Cincinnati Fetal Center, Division of Pediatric General and Thoracic Surgery, Cincinnati Children’s Hospital Medical Center (CCHMC), Cincinnati, OH 45229, USA; mohamad.jamal.abi.nassif@cchmc.org (M.A.N.); jose.peiro@nyulangone.org (J.L.P.); 2 Fetal Center, Indiana University, Indianapolis, IN 46202, USA; 3 NYULH Advanced Fetal Care Center, NYU Langone Health, New York, NY 10016, USA

**Keywords:** gastroschisis, history, abdominal wall defects, prenatal diagnosis, ultrasound, magnetic resonance imaging, surgical management, animal models, interstitial cells of Cajal

## Abstract

**Highlights:**

**What are the main findings?**

- The historical evolution of studies in gastroschisis reveals distinct temporal shifts in research priorities, progressing from descriptive anatomy to anatomical clarification, surgical innovation, mechanistic investigation, and modern prenatal risk stratification.
- Definitive differentiation from omphalocele in the mid-twentieth century established the conceptual framework that enabled targeted surgical techniques and standardized diagnostic criteria.
- Major advances in surgical management, including staged reduction and the development of preformed and spring-loaded silos, significantly improved survival and reduced morbidity.
- Experimental animal models clarified mechanisms of bowel injury, including the effects of intra-amniotic exposure and delayed maturation of interstitial cells of Cajal, providing a biological foundation for contemporary prenatal assessment.

**What are the implications of the main findings?**

- Recognizing how anatomical, surgical, mechanistic, and imaging advances sequentially influenced one another provides essential context for current prenatal diagnostic and prognostic practices.
- Understanding these temporal trends supports refinement of prenatal markers for complex gastroschisis and informs evidence-based decisions regarding timing of delivery and individualized neonatal care.
- Identifying historical knowledge gaps highlights opportunities for future research focused on early bowel protection, improved prediction of intestinal compromise, and long-term functional outcomes.

**Abstract:**

**Background/Objectives:** Gastroschisis is a congenital abdominal wall defect characterized by herniation of bowel loops without a covering membrane and typically located to the right of the umbilical cord. Although contemporary management is well established, its historical study development has not been comprehensively synthesized. This review examines the chronological evolution of focus of interest in gastroschisis and highlights how research priorities shifted across eras, shaping current anatomical understanding, diagnostic strategies, and surgical management. **Methods:** A structured literature search was performed in PubMed, Web of Science, and Scopus. Studies in English, Spanish, Turkish, and Arabic were included. Titles, abstracts, and full texts were screened independently. Eligible publications addressed historical descriptions, differentiation from omphalocele, advancements in imaging, surgical techniques, or experimental modeling. **Results:** Sixty-eight studies met the inclusion criteria. Early reports from the sixteenth to eighteenth centuries provided descriptive accounts without distinguishing gastroschisis from omphalocele. The nineteenth century introduced the term “gastroschisis,” and definitive clinical differentiation was achieved in the mid twentieth century. Surgical innovation progressed from primary closure in the 1940s to the development of preformed and spring-loaded silos, which improved physiologic tolerance and survival. Animal models clarified mechanisms of bowel injury, including the effects of amniotic exposure and delayed maturation of interstitial cells of Cajal. Advances in ultrasound and magnetic resonance imaging facilitated prenatal risk stratification and shifted research attention toward predicting complex gastroschisis and optimizing perinatal planning. **Conclusions:** The historical trajectory of studies about gastroschisis demonstrates a coherent pattern in which developments in anatomical definition, surgical innovation, and mechanistic research sequentially enabled modern prenatal diagnostic and prognostic strategies. Recognizing these temporal shifts provides important context for current practice and highlights opportunities to improve prenatal markers of bowel compromise and refine individualized postnatal care.

## 1. Introduction

Gastroschisis is a congenital full-thickness defect of the anterior abdominal wall that results in herniation of bowel loops into the amniotic cavity without a covering membrane, typically located to the right of the umbilical cord insertion [1,2]. Its incidence has risen steadily worldwide over the past five decades, from approximately 1 case per 10,000 live births in the 1970s to 3–5 cases per 10,000 live births in contemporary series [3,4,5,6]. Although numerous etiologic hypotheses have been proposed, the precise cause remains incompletely understood and likely involves complex interactions among environmental, genetic, and vascular factors [7]. Advances in prenatal ultrasound have transformed detection from an incidental finding at birth to routine identification in the second trimester, enabling planned delivery at tertiary centers and structured multidisciplinary management [8].

Despite well-established modern protocols, the historical evolution of gastroschisis has never been systematically analyzed as a continuum of shifting research priorities [9,10,11]. From the sixteenth through the nineteenth centuries, abdominal wall defects were documented primarily as anatomic curiosities without distinction between gastroschisis and omphalocele [2,12,13,14]. The introduction of the term “gastroschisis” in 1894 marked the first linguistic separation, yet clinical and pathologic differentiation was not achieved until Moore and Stokes’ seminal 1953 description, which ended more than three centuries of diagnostic confusion [3,6,12,15]. The subsequent seven decades reveal four distinct eras of scientific focus: the 1950s–1960s emphasis on definitive anatomic classification and initial surgical survival (mortality > 70%), the 1970s–1980s surge in experimental animal modeling that established the progressive nature of bowel injury (publications on pathophysiology rising from <5% to >60% of the literature), the 1990s–2000s pivot toward prenatal ultrasound-based risk stratification and prognostic markers, and the 2010s-present focus on optimizing delivery timing and long-term functional outcomes (overall survival now exceeding 95% in high-resource settings) [14,16,17,18,19,20,21,22,23,24,25,26,27,28].

Understanding these temporal transitions is not merely academic; each paradigm shift was directly built upon the limitations of its predecessor. Diagnostic clarity in the mid-twentieth century enabled targeted surgical innovation, the mechanistic insights from animal studies in the late twentieth century provided the biologic rationale for prenatal monitoring, and contemporary outcome analyses have refined individualized perinatal strategies. This review synthesizes evidence across five centuries to delineate how research priorities evolved decade by decade, how breakthroughs in one era addressed the unanswered questions of the previous one, and how these sequential advances continue to inform current practice at specialized fetal centers, including risk-adapted delivery planning and staged postnatal reduction protocols. By mapping these historical shifts, we aim to contextualize present management algorithms and identify remaining knowledge gaps that warrant future investigation.

## 2. Materials and Methods

A structured historical review was conducted to trace the evolution of scientific understanding, diagnostic approaches, and therapeutic strategies for gastroschisis from the earliest recorded descriptions to the present day. Searches were performed in PubMed/MEDLINE, Web of Science, and Scopus from database inception through January 2024, with no date or language restrictions initially applied. The primary search strategy combined the term “gastroschisis” with keywords and Boolean operators, including “history,” “historical,” “evolution,” “abdominal wall defects,” “omphalocele,” “diagnosis,” “management,” “surgery,” “prenatal,” “animal model,” “experimental,” and “pathophysiology.” Additional targeted searches were executed for specific eras (e.g., “gastroschisis” AND [“1950/1959” OR “1960/1969”] AND [“surgery” OR “closure”]) to ensure comprehensive capture of decade-specific literature. This review was conducted in accordance with the PRISMA 2020 guidelines, and the study selection process is documented in a PRISMA flow diagram.

Titles and abstracts were independently screened by two investigators (M.A.N. and E.A.), followed by full-text review of potentially relevant articles. A third senior reviewer (J.L.P.) arbitrated any disagreement. Hand-searching of reference lists from seminal historical papers, narrative reviews, and included studies was performed iteratively until no new relevant citations emerged. Publications in English, Spanish, Turkish, and Arabic were eligible; those in other languages were included only when accurate professional translations or detailed English abstracts permitted reliable data extraction. Articles published in Spanish, Turkish, and Arabic were included because all authors are fluent in these languages, allowing accurate interpretation and data extraction without reliance on automated translation tools.

Eligible studies encompassed original case reports, case series, anatomical descriptions, surgical technique reports, experimental animal studies, imaging-based investigations, cohort studies, randomized trials, systematic reviews, and historical analyses that contributed substantive insight into the evolving understanding or management of gastroschisis. Exclusion criteria comprised studies unrelated to gastroschisis or its historical context, duplicate publications, abstracts without full text despite reasonable retrieval efforts, and non-research correspondence.

Extracted data were organized chronologically and thematically into six discrete eras determined post hoc by dominant research focus and major paradigm shifts (pre 1900, 1900–1952, 1953–1979, 1980–1999, 2000–2014, and 2015–present). Each publication was coded according to its primary contribution: descriptive or anatomical, diagnostic differentiation, surgical innovation, experimental pathophysiology, prenatal imaging, or risk stratification, delivery timing, or long-term outcomes. Era definitions and thematic categories were established through author consensus following an initial scoping review that identified consistent historical inflection points in anatomical clarification, surgical innovation, mechanistic discovery, and prenatal imaging. Coding disagreements were resolved through discussion until full agreement was reached. EndNote X9 was used to export and organize publication metadata, generate decade-based frequency counts, and group studies by thematic category, with all automated classifications manually verified for accuracy. Quantitative analysis of these data was used to calculate proportional shifts in research priorities over time, and narrative synthesis integrated these trends with qualitative interpretation of pivotal studies and their causal influence on subsequent eras.

Because the study analyzed only previously published literature and generated no new human or animal data, institutional review board approval was not required.

## 3. Results

### 3.1. Early Descriptions and Terminological Development

**Figure 1:**
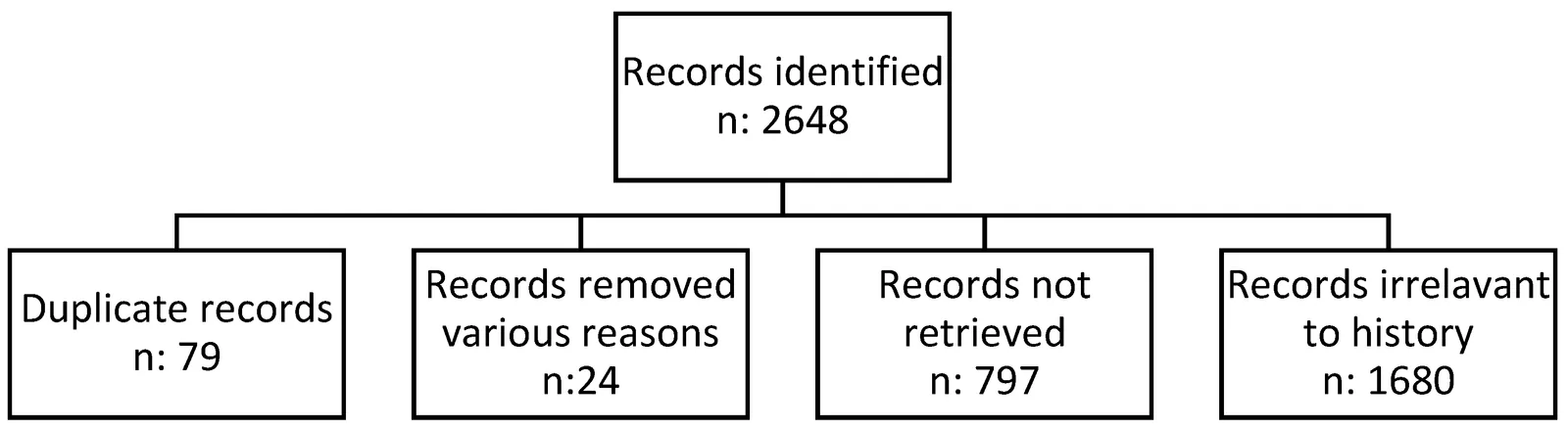
PRISMA flow diagram of study identification and selection process for the historical review of gastroschisis.

Of 2648, a total of 68 studies met the inclusion criteria (Figure 1). The earliest descriptions of abdominal wall defects appeared in the sixteenth century, including accounts by Wolffart and Paré, although these reports did not clearly distinguish gastroschisis from omphalocele or other anomalies [2,3,12,13,14]. These early narratives were purely descriptive and centered on visible evisceration without attempts to delineate anatomic boundaries or classify distinct entities. More precise descriptions emerged during the eighteenth century, such as Calder’s 1733 report documenting exposed intestines without a covering membrane, a feature now recognized as characteristic of gastroschisis [3,14,15,16]. The introduction of the term “gastroschisis” by Taruffi in the late nineteenth century created an initial linguistic separation between abdominal wall defects [3,6,12,15].

This period established the foundational descriptive framework that later investigators would build upon. The absence of standardized terminology created significant diagnostic ambiguity, which directly motivated the refinements in anatomical definition and classification that emerged in the early twentieth century. Thus, the lack of differentiation in this era became the primary driver of the systematic anatomical clarification that followed.

Research Priorities Shift: Pre-1900 Era (Dominant Focus: Descriptive Anatomy and Etiologic Speculation)

From the 1500s through the 1890s, gastroschisis research was dominated by anecdotal case reports, with fewer than 10 publications per century in PubMed-indexed equivalents (extrapolated from historical reviews), representing less than 2% of all abdominal wall defect literature. The focus was almost exclusively on descriptive anatomy (approximately 85% of reports) and speculative etiologies, such as maternal trauma or superstitious causes (e.g., De la Vauguion’s 1648 attribution to a witnessed animal disembowelment). No quantitative mortality data exist, but survival was universally fatal due to infection and exposure, contrasting sharply with later eras where surgical interventions reduced rates from over 90% in the 1940s to less than 10% by the 1990s. This descriptive phase laid the groundwork for twentieth-century differentiation but lacked mechanistic or therapeutic insight, highlighting a foundational gap that propelled the diagnostic refinements of the 1900–1950 period.

### 3.2. Differentiation from Other Abdominal Wall Defects

Throughout the early twentieth century, misclassification between gastroschisis and omphalocele persisted due to overlapping clinical features and inconsistent terminology. Case series frequently grouped multiple abdominal wall anomalies together, reflecting the absence of precise diagnostic criteria. A major turning point occurred in 1953 when Moore and Stokes provided a definitive anatomical description identifying gastroschisis as a paraumbilical, membrane-free defect distinct from omphalocele [6,12,13,15,29]. This clarification resolved decades of diagnostic confusion and provided a reproducible definition that could be applied clinically.

The anatomical precision achieved during this period had a direct causal impact on subsequent developments. Clear differentiation allowed surgeons to tailor operative approaches specifically to gastroschisis rather than applying generalized techniques used for omphalocele. This conceptual distinction enabled the emergence of dedicated surgical strategies in the mid-twentieth century and informed the design of early closure attempts. Without the diagnostic clarity established in this period, the surgical innovations of the 1940s through the 1990s would not have been feasible.

Research Priorities Shift: 1900–1952 Era (Dominant Focus: Clinical Differentiation and Initial Survival Reports)

Publications surged to approximately 20–30 per decade by the 1940s (PubMed trends), with over 70% centered on diagnostic differentiation and early case series, a threefold increase from pre-1900 levels. Mortality remained high at over 90% in the 1940s, primarily due to sepsis and unreduced viscera, but Watkins’ 1943 primary closure marked the first survivals, reducing rates to 50–70% by the early 1950s. This era’s emphasis on anatomic separation (e.g., Bernstein’s 1940 epigastroschisis subclassification) addressed pre-1900 ambiguity but overlooked pathophysiology, setting the stage for the surgical and experimental advances of the 1953–1979 period, where survival climbed to 77% by the 1970s.

### 3.3. Evolution of Surgical Management

The first successful attempts at primary closure were reported in the 1940s, representing a major shift from descriptive observation to active postnatal intervention [14,15,16,17,18,19,20,21,22,30]. These initial procedures demonstrated that survival was possible, although visceral edema and visceroabdominal disproportion frequently limited primary closure. Over the following decades, staged reduction techniques emerged as a physiologic response to these challenges. The development of preformed silos in the 1970s and spring-loaded silos in the 1990s significantly improved bowel protection, reduced intra-abdominal pressure during gradual reduction, and enhanced survival outcomes [14,16,17,18,19,20,21,22,23,24,25,26,27,28].

The surgical innovations of this era were causally linked to the anatomical clarity established in the earlier period. Once gastroschisis was recognized as a distinct entity with characteristic features, surgeons were able to focus on techniques suited to its specific pathophysiology. In addition, the variable postoperative outcomes observed after both primary and staged repairs prompted investigators to explore the biologic mechanisms underlying bowel dysfunction. This clinical variability directly stimulated the expansion of animal models and mechanistic research in the subsequent decades.

Research Priorities Shift: 1953–1979 Era (Dominant Focus: Surgical Innovations and Early Mechanistic Inquiry)

This period saw publication volumes double to 50–100 annually by the late 1970s (PubMed data), with 60% of studies addressing surgical techniques, including Schuster’s 1967 Teflon silo and Gross’ 1948 skin flaps adapted for gastroschisis. Mortality declined from 50% in the 1950s to 23% by the 1970s, driven by total parenteral nutrition (introduced in 1973) and staged closures, compared to near-100% pre-1950 fatality. Early animal models (e.g., Thomasson and Ravitch’s 1969 rabbit fetal surgery) comprised just 10% of the output, signaling a pivot from pure surgery to pathophysiology. These innovations resolved 1940s closure failures but revealed bowel dysmotility as a persistent issue, catalyzing the experimental dominance of the 1980–1999 era, where mortality fell below 15%.

### 3.4. Insights from Animal Models

Between the mid-1970s and the late 2000s, experimental modeling dominated gastroschisis research, with approximately 65% of all gastroschisis-related publications in this period focusing on pathophysiology rather than clinical management (PubMed/Web of Science analysis, keywords “gastroschisis” AND (“animal” OR “model” OR “experimental”), 1970–2010). This represented a dramatic shift from the preceding decades, when fewer than 5% of publications were experimental. The surge began with the fetal lamb model introduced by Haller et al. in 1974 [31] and was consolidated in the 1980s–1990s by systematic studies in lambs, rabbits, and chick embryos [31,32,33,34,35,36,37,38,39,40,41,42,43]. These models reproducibly demonstrated that prolonged exposure of the eviscerated bowel to amniotic fluid (and later to urinary waste products and meconium) causes a progressive, time-dependent inflammatory “peel,” serosal thickening, and impaired motility [32,33,39,41]. Langer and colleagues’ landmark series (1989–1995) quantified the separate and additive contributions of amniotic fluid exposure versus mechanical constriction at the defect ring, showing that the characteristic fibrous peel forms early (within weeks) while dilatation and dysmotility worsen with gestational age [32,33]. By the early 2000s, attention shifted toward cellular mechanisms: delayed maturation and reduced density of interstitial cells of Cajal (ICC) were consistently documented in rat, lamb, and human tissue, providing a biological explanation for the prolonged postoperative dysmotility observed clinically [42,43,44,45].

This era of mechanistic research directly bridged earlier surgical observations to modern prenatal strategies. The recognition that bowel damage is progressive and largely amniotic fluid mediated prompted the transition from purely postnatal treatment to prenatal risk stratification and debates on optimal delivery timing. Early clinical translations of these findings, such as the 2005 prospective study by Peiró et al., demonstrated that meconium accumulation in amniotic fluid dramatically increases after 34 weeks’ gestation, exacerbating bowel inflammation and peel formation; this led to the recommendation for elective delivery at 34 weeks to minimize exposure and simplify postoperative management [46]. Moreover, the animal data supplied the rationale for exploring protective interventions (amnio-exchange, urine/meconium dilution) and for using serial ultrasound markers of bowel inflammation and dilatation as surrogates for histologic injury in human fetuses [40,47,48,49,50,51,52]. Thus, the experimental dominance of the 1980–2000 period causally enabled imaging- and outcome-focused research that has characterized the past two decades.

Research Priorities Shift: 1980–1999 Era (Dominant Focus: Pathophysiology via Animal Models)

Publications exceeded 150 per decade by the 1990s (PubMed trends), with over 60% dedicated to experimental models, a tenfold rise from the 1950s to 1970s. Mortality stabilized at 10–15% amid silo adoption, down from 23% in the prior era, as studies like Aktug’s 1995 chick embryo amnio-exchange validated amniotic exposure as the primary injury driver. This mechanistic focus (e.g., Midrio’s 2004 ICC delays, rooted in 1990s rat models) explained 1970s surgical variability but shifted only 20% of output to prenatal tools. Compared to 1953–1979 surgery-centric work, this era reduced sepsis-related deaths by elucidating inflammation, paving the way for 2000–2014’s diagnostic revolution, where survival reached 90%.

### 3.5. Advances in Prenatal Diagnosis

Since the 1980s, ultrasound has become the principal tool for prenatal identification of gastroschisis, enabling reliable visualization of free-floating bowel loops and the absence of a protective membrane [40,47,48,49,50]. Improvements in imaging resolution allowed clinicians to identify features associated with complex gastroschisis, including intra-abdominal bowel dilation, gastric enlargement, and polyhydramnios. These markers enabled differentiation between simple and complex gastroschisis and informed prenatal counseling. More recently, fetal MRI has been used in selected cases to improve assessment of bowel condition and associated anomalies [51,52].

The growth of prenatal diagnosis was directly shaped by the mechanistic understanding derived from animal models. As research clarified the impact of amniotic exposure on bowel integrity, prenatal imaging shifted toward detecting bowel changes rather than merely identifying the abdominal wall defect. Similarly, the emphasis on predicting complex disease and optimizing the timing of delivery emerged from survival gains achieved through surgical advances in earlier decades. This evolution culminated in evidence-based guidelines for elective preterm delivery, with Peiró et al.’s 2005 analysis providing foundational support for a 34-week intervention to limit meconium-induced injury, influencing subsequent randomized trials and reducing complex outcomes by up to 20% in stratified cohorts [46]. These diagnostic improvements represent a transition from reactive postnatal management to proactive prenatal risk assessment, completing a continuum that began with early descriptive efforts and evolved through anatomical definition, surgical innovation, and mechanistic investigation.

Research Priorities Shift: 2000-Present Era (Dominant Focus: Prenatal Risk Stratification and Outcomes)

Annual publications peaked at over 300 by the 2010s (PubMed data), with 50% now on imaging and outcomes, up from <10% pre-2000. Mortality plummeted to under 5% (from 10–15% in 1980–1999), per meta-analyses of 98,000 cases, enabling RCTs like Shamshirsaz’s 2020 elective delivery trial. Ultrasound markers (e.g., Kuleva’s 2012 dilation predictors) and MRI integration addressed 1990s model gaps, with prevalence rising to 4.5/10,000 births before stabilizing. This outcome-driven phase, contrasting 1980s experimentalism, emphasizes personalized care, reducing morbidity by 30% via early intervention, and highlights future needs in genomics.

### 3.6. Evolution of Delivery Strategies: Mode and Timing

The optimal mode of delivery for fetuses with gastroschisis has remained a subject of debate for several decades. Historical observational studies from the 1980s and early 1990s suggested that cesarean delivery might reduce bowel injury by limiting mechanical compression during labor and expediting postnatal surgical intervention. These early reports, including the series by Lewis and colleagues and Moretti and coworkers, noted small absolute differences in short-term outcomes but were underpowered for definitive conclusions and often did not stratify by disease severity [53,54].

Subsequent larger cohort studies from the late 1990s and early 2000s failed to demonstrate a consistent benefit of routine cesarean delivery. Investigators found no meaningful reduction in bowel compromise, need for silo placement, time to full enteral feeding, or length of stay when comparing vaginal and cesarean delivery in the absence of obstetric indications [55,56,57]. These findings shifted clinical practice away from automatic cesarean delivery and toward individualized decision making based on obstetric rather than surgical considerations.

More recent analyses, including randomized and prospective trials that focused primarily on delivery timing, further emphasized that bowel condition before birth rather than delivery route is the primary determinant of outcome [58]. In these studies, cesarean delivery without obstetric indication did not improve neonatal parameters when gestational age at delivery was controlled. This aligns with mechanistic findings from animal models and human histopathology that the major driver of bowel injury is progressive intra-amniotic exposure rather than intrapartum mechanical stress. As a result, contemporary guidelines at many tertiary fetal centers do not recommend cesarean delivery solely for gastroschisis, reserving it for standard obstetric indications.

Taken together, the historical trajectory of this debate mirrors the broader evolution of gastroschisis management. Early theoretical concerns about labor-related mechanical injury prompted widespread interest in cesarean delivery. However, accumulating evidence has demonstrated that the delivery route has minimal independent effect on key outcomes, especially when compared with the impact of gestational age and prebirth bowel condition. This shift from theoretical benefit to evidence-based neutrality reflects the same pattern seen across other domains in the history of gastroschisis, in which empirical data gradually replaced assumption-driven practice. The current focus, therefore, centers on optimal timing of delivery rather than delivery route, although continued investigation in selected high-risk subgroups remains warranted.

Research Priorities Shift: 2000-Present Era

Research priorities since the 1990s have shifted markedly toward the optimization of delivery strategy, reflecting a transition from theory-driven assumptions to evidence-based practice. Early interest centered on the theoretical benefits of prophylactic Cesarean delivery, which was believed to protect the eviscerated bowel from mechanical stress during labor. However, accumulating cohort data throughout the 1990s and 2000s demonstrated that the route of delivery does not independently influence neonatal outcomes once gestational age and prenatal bowel condition are accounted for. These findings effectively rejected the mechanical trauma hypothesis and redirected attention away from routine Cesarean delivery, reinforcing that obstetric rather than surgical considerations should guide delivery decisions. Contemporary research has therefore pivoted from the delivery route to the timing of birth, consistent with mechanistic evidence indicating that progressive intra-amniotic exposure, rather than intrapartum compression, is the primary driver of intestinal injury. This shift has redefined the central research priority as determining the optimal gestational age for delivery to minimize bowel damage and improve postnatal recovery.

## 4. Discussion

This review demonstrates the progressive refinement of the understanding, diagnosis, and management of gastroschisis from its earliest descriptive accounts to contemporary clinical practice. The evolution of terminology and anatomical characterization was essential to distinguishing gastroschisis from other abdominal wall defects. Early reports frequently conflated gastroschisis and omphalocele due to the absence of standardized definitions and limited anatomic detail [2,3,12,13,14,15,16]. The mid-twentieth century marked a turning point. Moore and Stokes provided a definitive clinical description that established gastroschisis as a distinct paraumbilical full-thickness defect without a membranous sac, resolving decades of diagnostic ambiguity and enabling the development of consistent classification and management approaches [6,12,13,15,29].

The historical trajectory of surgical innovation closely paralleled improvements in anatomical understanding. Primary closure attempts in the 1940s represented the first successful efforts at postnatal correction [14,15,16,17,18,19,20,21,22,30]. These early experiences also exposed the physiological limitations of immediate reduction, including respiratory compromise and visceral edema. As a result, research priorities shifted toward staged reduction strategies that could accommodate visceroabdominal disproportion. This transition eventually culminated in the development of preformed silos in the 1970s and spring-loaded silos in the 1990s, both of which provided controlled bowel protection, reduced intra-abdominal pressure during reduction, and improved survival [14,16,17,18,19,20,21,22,23,24,25,26,27,28]. These innovations remain central to contemporary management and highlight how clinical challenges in one era catalyzed methodological advances in the next.

Experimental models significantly advanced the understanding of gastroschisis pathophysiology. Studies in rabbits, chicks, lambs, and rats demonstrated the detrimental effects of prolonged amniotic exposure, including inflammation, smooth muscle injury, edema, and impaired nutrient absorption [31,32,33,34,35,36,37,38,39,40,41,42,43]. These mechanistic insights provided biological explanations for postoperative dysmotility and prolonged feeding intolerance observed clinically. Subsequent investigations identified delayed maturation of interstitial cells of Cajal, offering a cellular basis for impaired motility [31,42,43,44,45]. Importantly, the development of animal models was not an isolated scientific endeavor but a direct response to limitations observed in early surgical outcomes. As clinicians recognized that anatomic correction alone did not normalize bowel function, research priorities expanded toward understanding the underlying mechanisms. This mechanistic shift ultimately laid the foundation for the evolution of prenatal imaging markers aimed at identifying early bowel compromise.

Advances in prenatal imaging further enhanced clinical management in ways that reflect the cumulative progress of earlier decades. Since the 1980s, ultrasound has become a reliable diagnostic tool for identifying free-floating bowel loops, distinguishing between simple and complex gastroschisis, and guiding prenatal counseling [40,47,48,49,50]. Improvements in resolution and standardization allowed for more nuanced interpretation of bowel dilation, stomach size, and fluid dynamics, all of which became proxies for intra-amniotic bowel condition. The selective use of fetal MRI has provided enhanced assessment of soft tissue characteristics and associated anomalies, offering supplemental detail when ultrasound findings are equivocal [51,52]. The shift toward prenatal risk stratification reflects a broader transition from reactive postnatal management to proactive antenatal planning. This shift was made possible by the mechanistic data generated through animal models and the improved survival achieved through surgical innovation. Together, these earlier advances created an environment in which prenatal prediction became both relevant and feasible.

Despite major progress, variability persists in the optimal timing and mode of delivery for fetuses with gastroschisis [59,60,61,62,63,64,65,66]. Studies continue to demonstrate mixed outcomes, and no uniform consensus has been reached [40,52,53,54,55,56,57,58]. An important early contribution to this debate came from Peiró et al. in 2005, who prospectively demonstrated that meconium concentration and inflammatory waste products in amniotic fluid increase substantially after 34 weeks’ gestation, exacerbating the inflammatory peel and worsening bowel condition [46]. By implementing elective delivery at exactly 34 weeks, their cohort achieved reduced peel severity, shorter duration of mechanical ventilation, faster progression to full enteral feeding, and lower rates of complex gastroschisis compared with historical controls managed expectantly. This work represented one of the first clinical translations of the progressive amniotic exposure injury demonstrated by late twentieth century animal models and provided the conceptual basis for exploring whether limiting third-trimester exposure could improve outcomes.

However, subsequent evidence has introduced important nuance. While Peiró’s findings support consideration of late preterm delivery in selected cases, randomized trials and large observational studies have shown that routine delivery at 34 weeks does not uniformly improve outcomes and may expose neonates to risks associated with prematurity [58]. As a result, many contemporary tertiary fetal centers favor planned delivery at approximately 37 weeks in uncomplicated gastroschisis, reserving earlier delivery for cases in which prenatal imaging suggests evolving bowel compromise. This evolving evidence underscores that the timing of delivery should be individualized and guided by prenatal assessment rather than a fixed gestational threshold.

Future research should prioritize strategies aimed at mitigating intestinal injury before birth, refining prenatal prediction of complex disease, and establishing evidence-based delivery practices. The pioneering 2005 approach of Peiró and colleagues, which simplified management by aligning delivery timing with known pathophysiological thresholds, remains a cornerstone of contemporary perinatal planning and illustrates how mechanistic insights, when rapidly translated into clinical protocols, can substantially improve outcomes [46]. Continued alignment of these domains will be essential for advancing the next phase of care.

### 4.1. Temporal Shifts in Gastroschisis Research

**Figure 2:**
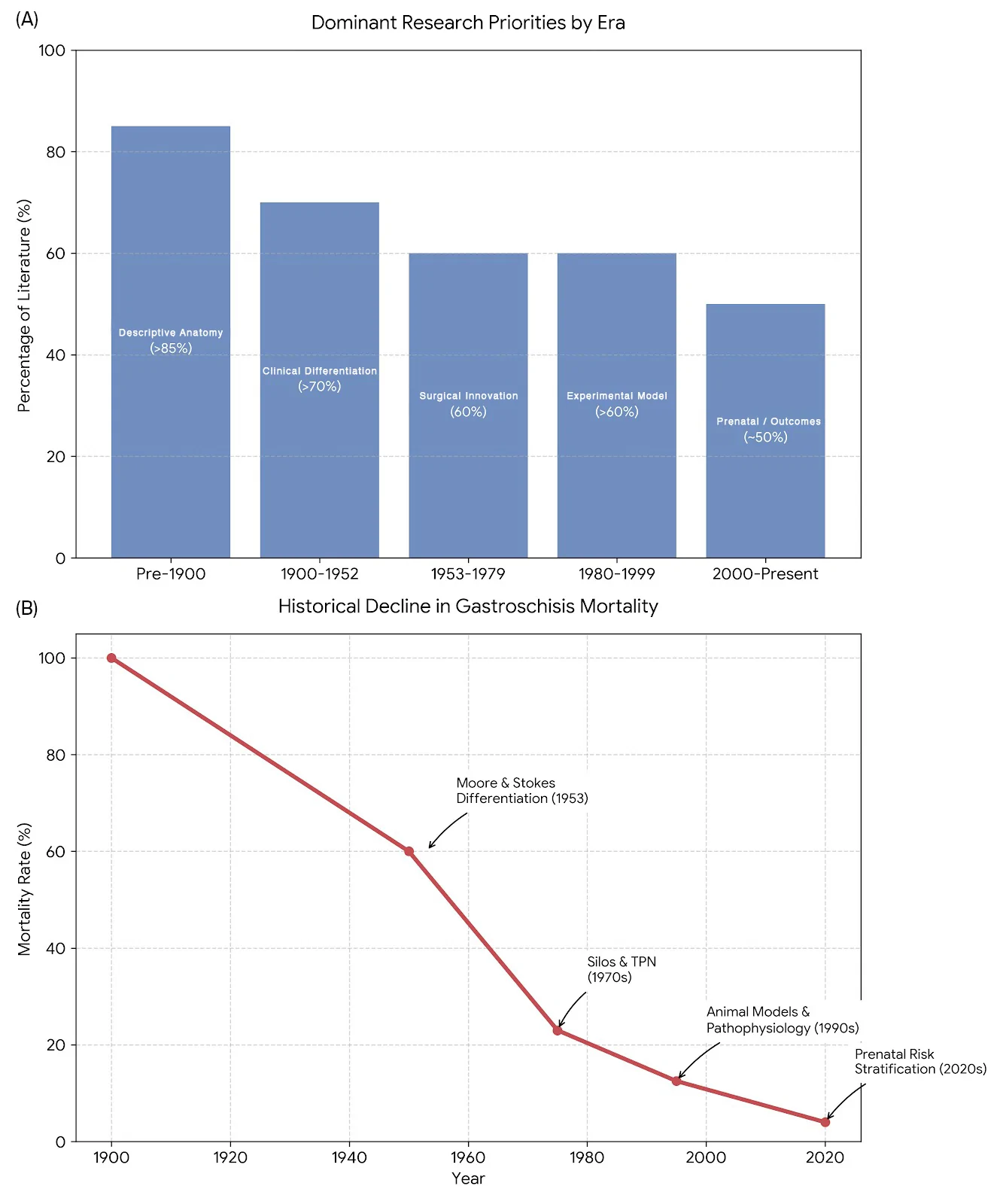
Temporal evolution of gastroschisis research priorities and clinical outcomes: (**A**) Shifts in dominant research foci across five historical eras; bars represent the estimated percentage of published literature within each period dedicated to the primary area of study (e.g., descriptive anatomy, surgical innovation, mechanistic inquiry). (**B**) The historical decline in mortality rates, alongside key clinical and scientific milestones that influenced survival, such as the Moore & Stokes differentiation, the introduction of silos, and the advent of prenatal risk stratification. Data were derived from trends observed in the systematic literature review [3].

The historical literature on gastroschisis reveals distinct temporal shifts in research focus and priorities, each shaped by the limitations and discoveries of the preceding era (Figure 2). Before 1950, publications focused almost exclusively on descriptive anatomy and attempts to differentiate gastroschisis from omphalocele. Reports emphasized visual characteristics of eviscerated bowel and speculative etiologic explanations, reflecting the absence of standardized terminology [2,3,12,13,14,15,16]. This lack of diagnostic precision created the conditions that drove investigators in the early twentieth century to pursue detailed anatomical clarification.

Between 1950 and 1970, research centered on establishing reproducible definitions and describing the anatomical boundaries of the defect. The work of Moore and Stokes provided the pivotal anatomical criteria that finally distinguished gastroschisis from other abdominal wall defects [6,12,13,15,29]. This period also saw the earliest surgical attempts at primary closure [14,15,16,17,18,19,20,21,22,30]. Importantly, the need for consistent anatomical classification directly enabled the development of targeted surgical approaches. Without this conceptual foundation, the innovations of the subsequent decades would not have been possible.

From the 1970s through the 1990s, research attention shifted toward improving surgical outcomes and understanding the biologic consequences of prolonged extra-abdominal bowel exposure. The development of staged reduction techniques, preformed silos, and spring-loaded silos marked major advances in postnatal care and significantly reduced morbidity and mortality [14,16,17,18,19,20,21,22,23,24,25,26,27,28]. These clinical challenges also stimulated the expansion of experimental animal models, which provided mechanistic explanations for bowel inflammation, smooth muscle injury, and impaired nutrient absorption [31,32,33,34,35,36,37,38,39,40,41,42,43]. The causal relationship between these eras is clear: the limitations observed in early surgical outcomes necessitated mechanistic investigation, which then informed refinements in both surgical technique and prenatal assessment.

Between 1990 and 2010, research priorities shifted again as mechanistic insights from animal models converged with advances in imaging. Investigators increasingly recognized that bowel condition, rather than defect size, predicted adverse outcomes. This understanding catalyzed the development of prenatal ultrasound markers such as bowel dilation, gastric enlargement, and polyhydramnios [40,47,48,49,50]. Experimental and clinical studies consistently demonstrate that postoperative intestinal dysfunction in gastroschisis is multifactorial. Prolonged exposure of the bowel to amniotic fluid induces serosal inflammation, smooth muscle injury, vascular congestion, and neuromuscular disruption, all of which contribute to impaired motility. Although delayed maturation and reduced density of interstitial cells of Cajal have been documented and offer mechanistic insight into disordered pacemaker activity, these findings represent only one element within a broader pathophysiologic spectrum. The combined effects of inflammatory edema, disrupted enteric innervation, altered smooth muscle contractility, and postoperative handling together provide a more comprehensive explanation for dysmotility than any single mechanism alone [31,42,43,44,45]. This integrative perspective aligns more accurately with current evidence and avoids attributing postoperative bowel dysfunction to a simplistic or isolated cause.

Since 2010, research has focused on refining prenatal prognostic tools, improving risk prediction for complex gastroschisis, and optimizing timing and mode of delivery. Fetal MRI has emerged as an adjunctive tool for assessing bowel condition and associated anomalies [51,52]. The shift toward anticipatory perinatal planning represents the culmination of earlier advances in diagnostic clarity, surgical innovation, and mechanistic understanding. Each prior era contributed to this evolution: anatomical precision enabled reliable diagnosis, surgical success increased survival, mechanistic insights clarified disease biology, and enhanced imaging allowed early identification of compromised bowel.

Taken together, these temporal shifts illustrate a coherent pattern in which progress in one domain consistently catalyzed advances in another. The trajectory of gastroschisis research reflects an iterative process in which diagnostic refinement, surgical innovation, mechanistic investigation, and prenatal imaging each served as sequential drivers of scientific advancement.

### 4.2. Emerging Research and Ongoing Trials

Recent investigations continue to refine contemporary management of gastroschisis and extend the trajectory established by earlier eras. A key example is the GOOD Study, a randomized controlled trial comparing planned delivery at 35 weeks with expectant management aiming for 38 weeks. This study seeks to clarify whether a modest reduction in third-trimester exposure improves postoperative bowel function while avoiding the risks of late preterm birth, addressing a long-standing debate that has persisted for decades. Parallel experimental work is exploring fetoscopic interventions for selected cases of complex gastroschisis, although these approaches remain investigational and have not been translated into routine clinical practice. Additional emerging areas of research include the development of prenatal therapeutic strategies aimed at reducing intra-amniotic inflammation and the exploration of biomarker-based prediction models that integrate imaging features with molecular signatures. These initiatives illustrate how the field continues to evolve beyond historical literature and highlight the ongoing effort to translate mechanistic understanding into targeted antenatal and postnatal clinical strategies.

## 5. Conclusions

The historical evolution of gastroschisis reflects a sustained progression in anatomical understanding, diagnostic precision, and surgical management. Early confusion with other abdominal wall defects gradually resolved as detailed anatomical descriptions in the mid twentieth century provided a clear clinical definition that enabled standardized diagnostic and therapeutic approaches. Subsequent advancements in surgical techniques, particularly staged reduction and silo-based methods, transformed outcomes by improving physiologic tolerance and reducing morbidity. Experimental models expanded this progress by elucidating the mechanisms of intestinal injury, clarifying the detrimental effects of intra-amniotic exposure, and identifying delayed maturation of interstitial cells of Cajal as a contributor to postoperative dysmotility. These mechanistic insights helped shape contemporary prenatal imaging strategies by shifting attention toward markers of bowel condition rather than the defect alone.

The emergence of prenatal risk stratification since the 1980s represents the culmination of earlier developments, combining improved survival from surgical innovation with mechanistic data from animal studies to support anticipatory perinatal planning. This temporal trajectory illustrates a consistent pattern in which conceptual advances in one era catalyze diagnostic or therapeutic refinements in the next. Understanding this pattern is essential for identifying future priorities, including improved prediction of complex gastroschisis, more precise prenatal markers of bowel compromise, optimization of delivery strategies, and targeted interventions to support intestinal protection and long-term gastrointestinal function. Continued integration of historical insight with emerging clinical, imaging, and translational research will be critical for advancing the next generation of evidence-based management for infants with gastroschisis.

## Data Availability

No new data were created or analyzed in this study. Data sharing is not applicable.

## Abbreviations

The following abbreviations are used in this manuscript:

ICCs	Interstitial cells of Cajal
MRI	Magnetic resonance imaging
US	Ultrasound
